# Chronic cortisol exposure in early development leads to neuroendocrine dysregulation in adulthood

**DOI:** 10.1186/s13104-020-05208-w

**Published:** 2020-08-03

**Authors:** Ellen I. Hartig, Shusen Zhu, Benjamin L. King, James A. Coffman

**Affiliations:** 1 MDI Biological Laboratory, Salisbury Cove, Maine, USA; 2 Graduate School of Biomedical Sciences and Engineering, University of Maine, Orono, ME USA; 3 Department of Molecular and Biomedical Sciences, University of Maine, Orono, ME USA

**Keywords:** Glucocorticoids, Cortisol, Early life stress, Developmental programming, Epigenetic

## Abstract

**Objective:**

Chronic early life stress can affect development of the neuroendocrine stress system, leading to its persistent dysregulation and consequently increased disease risk in adulthood. One contributing factor is thought to be epigenetic programming in response to chronic cortisol exposure during early development. We have previously shown that zebrafish embryos treated chronically with cortisol develop into adults with constitutively elevated whole-body cortisol and aberrant immune gene expression. Here we further characterize that phenotype by assessing persistent effects of the treatment on cortisol tissue distribution and dynamics, chromatin accessibility, and activities of glucocorticoid-responsive regulatory genes *klf9* and *fkbp5*. To that end cortisol levels in different tissues of fed and fasted adults were measured using ELISA, open chromatin in adult blood cells was mapped using ATAC-seq, and gene activity in adult blood and brain cells was measured using qRT-PCR.

**Results:**

Adults derived from cortisol-treated embryos have elevated whole-body cortisol with aberrantly regulated tissue distribution and dynamics that correlate with differential activity of *klf9* and *fkbp5* in blood and brain.

## Introduction

Epidemiological studies have shown that early life stress (ELS) can increase later life risk of developing a variety of physical and mental health problems, many of which are linked to chronic inflammation [1–3]. This long-term ‘developmental programming’ has been hypothesized to in part be an epigenetic effect of chronic exposure to elevated cortisol levels [4, 5]. Consistent with this, chronic glucocorticoid (GC) exposure, such as occurs with chronic stress, genetic diseases such as Cushing’s syndrome, or with extended GC therapy, promotes development of metabolic and inflammatory disease [6]. Developmental programming in response to ELS can be adaptive to the extent that it tunes the responsiveness of the stress system to the stressfulness of the environment, but can be maladaptive when there is a mismatch between the adult environment and that which was encountered during earlier development [1, 7]. The cost of such programming can be an increased allostatic load that promotes unhealthy aging [8].

Zebrafish are emerging as an excellent model system for experimental studies of stress-induced developmental programming [9–18]. We recently showed that zebrafish embryos treated chronically with 1 μM cortisol develop into adults that maintain elevated whole body cortisol levels and aberrantly express pro-inflammatory genes, with higher basal expression levels in peripheral tissues but a blunted response to tailfin injury or lipopolysaccharide injection [13], indicating that the treatment has long-term effects on the neuroendocrine stress axis and GC-regulated gene expression. Those results are extended by the experiments reported here.

## Main text

### Results

#### Adults derived from cortisol-treated embryos display aberrant cortisol tissue distribution and dynamics

**Fig. 1:**
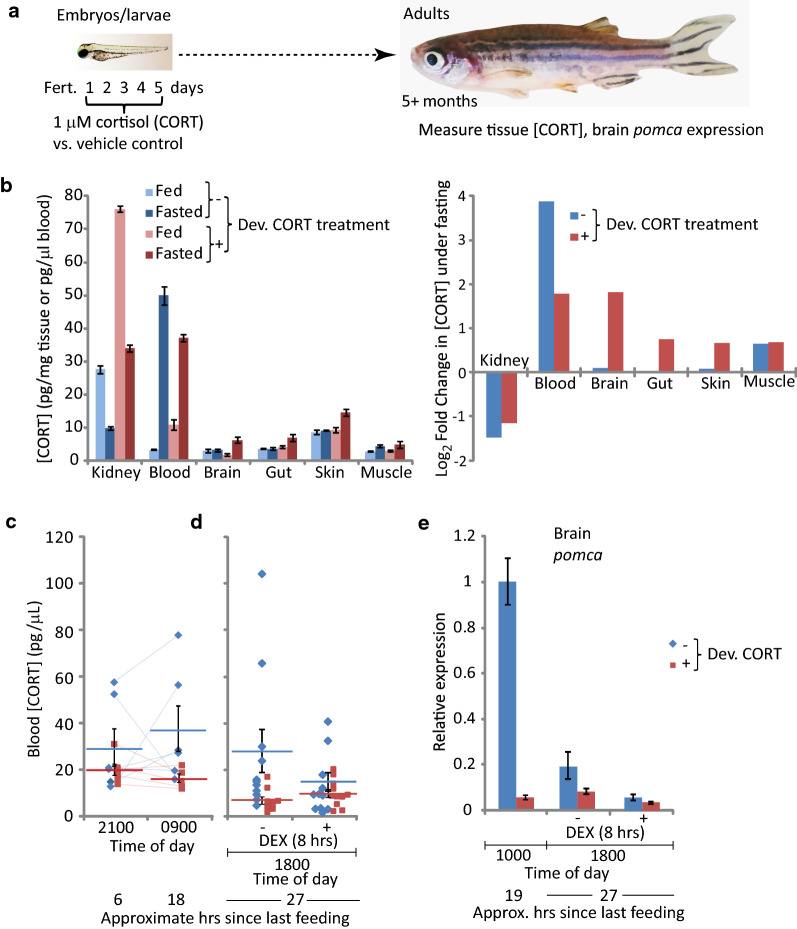
Embryos treated chronically with cortisol develop into adults with aberrant cortisol levels, tissue distribution, and dynamics. **a** Schematic of experimental design; **b** Cortisol levels in different tissues of fed and fasted (24 h) fish (left panel) and the inferred change in those levels induced by fasting (right panel). Each measurement was taken from pooled tissues of 6 fish, with equal numbers of males and females. Error bars are the standard deviations of technical replicates. **c** Cortisol levels in blood draws from single fish at night and the following morning, with the amount of time since the last feeding indicated. Thin lines between data points indicate repeat measurements from the same fish. Averages ± standard errors of the mean (SEM) are also shown. **d** Fasting cortisol levels in blood of individual fish (data points) following 8 h exposure to 1 μM dexamethasone (DEX) or vehicle. The averages ± SEM for each group are also indicated. The fact that the levels are somewhat lower than those shown in (**b**) may reflect circadian fluctuation (these samples were taken in late afternoon whereas those shown in panel (**b**) were taken mid-morning, ~ 8 h earlier). **e** Relative expression of *pomca* in brain tissue in the late morning, as well as later the same day following 8 h exposure to DEX or vehicle. The bars indicate averages ± the standard deviation of 3 qPCR readings (technical replicates). For each measurement brain tissue was pooled from 5–6 fish, mixed males and females in equivalent proportions within each comparison group (controls 1 male and 4–5 females; cortisol-treated 2 males and 3 females)

Zebrafish embryos were treated with cortisol or vehicle as described previously [13], then raised to adulthood (5 + months), at which time cortisol levels in different tissues were measured in fish that had recently been fed or that had been fasted for 24 h (Fig. 1a, b). As we observed previously [13], the fish in the cortisol treated group had higher whole-body cortisol levels than their untreated siblings, which in fed fish correlated with higher levels in kidney (which when dissected out likely contains the cortisol-producing interrenal gland) and blood (Fig. 1b). However, in fish that had been fasted for 24 h, cortisol levels were lower in blood of the cortisol-treated group compared to controls, but higher in the brain, skin and gut (Fig. 1b). Plotting of the ratios of cortisol levels in fed and fasted fish reveals that while 24 h of fasting did not affect brain cortisol levels in control fish, it caused brain cortisol levels to quadruple in the treated fish (Fig. 1b, right panel). A three-way ANOVA (Additional file 1) showed significant variance in cortisol levels across different tissues is (p = 0.005), as well as a significant interaction effect for tissue type and fed state (p = 0.035). Post-hoc two-way ANOVA indicated that fed state significantly affected cortisol levels in kidney, blood, brain, and muscle (Additional file 1).

In multiple experiments, fasted adults derived from cortisol-treated embryos consistently displayed lower blood cortisol on average, with a compressed dynamic range compared to their control siblings (Fig. 1b–d). Stress-induced spikes in brain cortisol (as appears to occur in the treated fish in response to fasting, Fig. 1b) would be expected to downregulate the hypothalamus–pituitary–adrenal/interrenal axis via glucocorticoid receptor (GR)-mediated negative feedback. Consistent with this, in fasted fish derived from cortisol-treated embryos, blood cortisol levels were similar to those of their untreated siblings that had been exposed to Dexamethasone (Dex) for 8 h and were not further reduced by the Dex treatment (Fig. 1d). Moreover, brain expression of the ACTH-encoding gene *pomca*, a target of GR-mediated repression, was significantly lower in fasted fish derived from cortisol-treated embryos and is less affected by Dex (Fig. 1e). Altogether these data confirm our earlier finding that whole-body cortisol levels are elevated in adult fish derived from cortisol-treated embryos, and show further that cortisol is aberrantly regulated in those adults, with a compressed dynamic range in blood that correlates with an expanded dynamic range in brain compared to controls. One possible explanation for this is that the cortisol treatment may have persistent effects on the system of cortisol buffering and transport in plasma, for example by affecting the synthesis or metabolism of plasma cortisol-binding proteins that regulate steroid delivery to tissues [19].

#### Adults derived from cortisol-treated embryos differentially express the glucocorticoid-responsive regulatory genes klf9 and fkbp5 in blood and brain

**Fig. 2:**
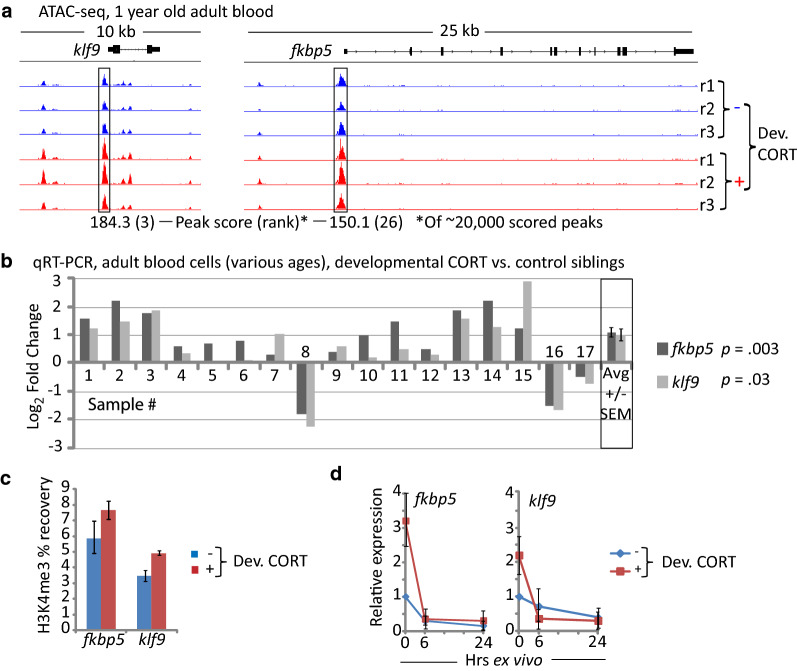
The regulatory genes *klf9* and *fkbp5* have higher on average activity in blood cells of adults derived from cortisol-treated embryos. **a** ATAC-seq peaks associated with *klf9* and *fkbp5*, from three biological replicates of each treatment (r1-r3). For each replicate sample blood was pooled from 6 individuals mixed sex with equal representation of males and females. **b** Relative expression of *klf9* and *fkbp5* in 17 blood samples of adults from different experimental cohorts of cortisol-treated embryos compared to their control siblings sampled at the same time. The averages ± SEM are also shown. For each experimental sample blood was pooled from 6 individuals of each group (control and treated), of mixed sex with equal representation of males and females. **c** ChIP-qPCR of H3K4me3 levels in the promoter regions of *klf9* and *fkbp5* from a single sample of blood pooled from 6 individuals (mixed sex, equal representation). The averages ± SD of three replicate qPCR measurements (technical replicates) are shown. **d** Relative expression of *klf9* and *fkbp5* in blood cells immediately after being drawn, and then after 6 or 24 h of ex vivo culture in the absence of cortisol. The plots represent the grand means ± the SEM of three biological replicates, each done on blood samples pooled from 6 individuals of mixed sex

To determine if developmental exposure to cortisol has persistent effects on gene activity at the level of chromatin we performed ATAC-seq [20, 21] on blood cells isolated from three independent replicates comparing ~ 1-year old adults derived from cortisol-treated embryos and their vehicle-treated (control) siblings. Each ATAC-seq peak was scored according to its size in cortisol-treated fish relative to controls, with higher scores indicating proportionally more sequence reads and hence chromatin with a more open configuration in the cortisol treated group. Of ~ 20,000 scored peaks, that with the 3^rd^ highest score encompassed the promoter of *klf9* (Fig. 2a; Table 1), a known GR target gene that functions as a feedforward regulator of GR signaling [22–26]. Several additional genes with high peak scores are also known GR targets in mammals, including *fkbp5* (Fig. 2a; Table 1), a clinically important feedback regulator of the GR [23, 27–30]. Three of the genes in the top 35 (*chac1*, *klf9*, and *fkbp5*) were found in our previous study to be highly upregulated in larvae chronically exposed to cortisol (Table 1). Interestingly, in a HOMER motif enrichment analysis [31] of the 251 peaks with scores  ≥ 100, four of the top ten scoring motifs were consensus binding sequences for krüppel-like factors, including Klf9 (Additional file 2: Figure S1).

**Table 1:** Top 35 ATAC-seq peaks in blood cells of 1-year old fish derived from cortisol-treated embryos

Peak Score	Nearest gene	Location	Distance to TSS
198.6	*chac1*^a^	Promoter-TSS	− 173
184.4	*nlk2*	Intergenic	− 1209
184.3	*klf9*^a,b^	Promoter-TSS	***− ***173
180.9	*spns2*	Intergenic	− 25,446
176.4	*mir7a*-*3*	TTS	685
173.1	*LOC564840*	Promoter-TSS	− 90
172.2	*stat3*	Promoter-TSS	− 148
171.3	*abhd12*	Intron (1 of 13)	765
170.7	*impdh2*	Promoter-TSS	− 218
169.2	*gpr146*	Promoter-TSS	− 234
168.7	*ppp2ca*	Promoter-TSS	− 92
167.4	*mtmr3*	Intron (1 of 19)	14,093
164.4	*tmem57b*	Promoter-TSS	− 181
163.9	*cep57l1*	Intergenic	− 3276
163.7	*sgk1*^b^	Promoter-TSS	− 128
163.6	*rab40b*	Promoter-TSS	− 90
158.6	*etnk2*	Intergenic	− 117,052
156.3	*dus1l*	Intergenic	− 4036
155.8	*txnipa*	Promoter-TSS	− 174
155.7	*glcci1*^b^	Promoter-TSS	− 153
154.9	*zranb1b*	Intergenic	− 9055
154.7	*mir144*	Promoter-TSS	− 502
154.6	*znf410*	Intergenic	− 15,262
154.3	*capgb*	Intergenic	− 16,617
150.9	*osgn1*	Promoter-TSS	− 117
150.1	*fkbp5*^a,b^	Promoter-TSS	− 159
149.8	*klf11a*	Promoter-TSS	− 85
149.8	*snrnp48*	Intergenic	− 8447
149.4	*gsk3b*	Promoter-TSS	− 182
148.7	*stat2*	Intergenic	− 1577
147.9	*cct4*	Intergenic	29,573
147.3	*gc2*	Intergenic	44,685
145.7	*hprt1l*	Promoter-TSS	− 91
145.2	*dyrk1b*	Intron (1 of 11)	42,251
144.0	*hlfa*	Intergenic	− 3552

^a^Found by RNA-seq to be strongly upregulated in cortisol-treated larvae at 5 dpf

^b^Known GR target in mammals

The ATAC-seq results suggest that adults derived from cortisol-treated embryos persistently upregulate the activities of both *klf9* and *fkbp5*, possibly in response to the persistently elevated cortisol levels. To further test this, quantitative reverse transcription and polymerase chain reaction (qRT-PCR) of both genes was carried out on blood cells of adults derived from cortisol-treated embryos and their vehicle-treated siblings. In 14 out of 17 independent blood cell samples, *klf9* and/or *fkbp5* expression was elevated in the cortisol-treated fish, both genes being overexpressed ~ twofold on average (Fig. 2b). The fact that *fkbp5* and *klf9* expression was elevated in most, but not all the samples from treated fish might relate to our observation that blood cortisol levels of the treated fish were lower than those of their untreated siblings after fasting (Fig. 1b–d), suggesting that the three samples that display lower *klf9* and *fkbp5* expression (8, 16, and 17) may have been recently stressed. Chromatin immunoprecipitation detected higher levels of H3K4 trimethylation in the promoter regions of both genes in blood cells of the treated fish (Fig. 2c), suggesting that the on-average elevated expression in those fish is transcriptional. This is likely a direct effect of elevated blood cortisol, as, when blood cells were cultured ex vivo the expression of both genes decreased over time (Fig. 2d).

In an initial experiment that surveyed the expression of 95 genes in adult brains, *fkpb5* and *klf9* stood out as the two that were most highly overexpressed in the cortisol-treated group compared to their control siblings (Additional file 3: Figure S2a). However, this result was not reproduced in measurements from additional experimental cohorts (Additional file 3: Figure S2b–f), suggesting that the fish used for the initial survey may have recently experienced a stressor that produced a spike in brain cortisol levels of the treated group (e.g., as observed with fasting; Fig. 1b). One strikingly consistent finding from the gene expression measurements made from both blood cells (Fig. 2b) and brain (Additional file 3: Figure S2) was that expression of *fkbp5* and *klf9* was highly correlated. This could simply reflect that they are both glucocorticoid-responsive genes, but it could also be indicative of additional regulatory interactions between the two genes. Finally, although most of the gene expression measurements were made from pools of mixed sexes containing equal numbers of matched-sized males and females, in an experiment where the sexes were segregated we found that brain expression levels for both genes were higher in males, and that the differential expression in treated and control groups was greater in males than in females (Additional file 3: Figure S2f), indicating that both genes are also regulated by sex.

### Conclusions

These results extend our earlier findings showing that in zebrafish, chronic glucocorticoid exposure during early development leads to persistent dysregulation of the neuroendocrine stress system and associated gene expression in adulthood. The data indicate that the neuroendocrine dysregulation occurs a multiple levels, including cortisol production (higher in treated fish), delivery of cortisol from circulation to tissues (lower blood retention in the treated fish after fasting, with concomitantly higher levels in brain and peripheral tissues), and activity of key genes that regulate GC signaling dynamics in receiving cells (*klf9* and *fkbp5*). These results underscore the relevance of zebrafish as a model organism for investigating the mechanisms underlying developmental programming and neuroendocrine dysregulation associated with ELS and chronic glucocorticoid exposure in humans.

### Methods

#### Zebrafish procedures

Wild-type zebrafish of the AB strain maintained in the MDI Biological Laboratory zebrafish facility as previously described [13] were used for all of the reported experiments. Cortisol treatment was carried out as previously described [13]. For the dexamethasone treatments adult zebrafish were removed from the re-circulating system and transferred into 1 L holding tanks containing either 1 µM dexamethasone or 2.5 ppm vehicle (DMSO) in system water. For tissue collection fish were euthanized in tricaine as previously described [13].

#### Ex vivo blood cell culture

Blood was collected by a nonlethal venous puncture under the operculum. 1µL samples of blood from 6 fish of mixed sex per condition were pooled in heparin (5 I.U./mL) in PBS, washed, and plated at a density of 1 million cells/mL in supplemented L-15 media (l-glutamine, 10% FBS, 25 mM HEPES, 2.2 g/L sodium bicarbonate, Primocin). Cells were cultured at 28° C in 5% CO_2_. Blood cell viability was assessed by cell count with a hemocytometer. Zebrafish blood cells were determined to be stable in culture for at least 5 days.

#### Cortisol measurements

Cortisol was measured by ELISA as previously described [13], using dissected tissues or blood samples. In some cases, blood was collected by nonlethal venous puncture under the operculum, allowing repeated measurements (e.g. as in Fig. 1c).

#### ATAC-seq

ATAC-seq libraries from blood cells (~ 200,000 cells per sample) from three biological replicates of adults derived from cortisol-treated embryos and their control (vehicle-treated) siblings (10, 11, and 12 months old) were constructed and sequenced and the sequence reads processed as described in the Additional file 4: Methods [20, 21].

#### RNA isolation and quantitative reverse transcription—polymerase chain reaction qRT-PCR

Total RNA was isolated from blood cells or dissected brain tissue using Trizol and ethanol precipitation. qRT-PCR was performed and the data quantified by the ddCt method as previously described [13].

#### Chromatin immunoprecipitation (ChIP)

ChIP was performed on formaldehyde-crosslinked chromatin from blood cells, essentially using the procedure described by Lindeman et al. [32] an anti-H3K4me3 antibody (Abcam Cat. No. Ab8580) (detailed ChIP protocol provided on request). Input and immunoprecipitated DNA samples were analyzed by qPCR with Sybr Green Fastmix. The primers were targeted to promoter regions of *klf9* (CACATCGACACCGCCTTCATCTG; ATCTTGAACCTCGCCGCTGATTG) and *fkbp5* (ACACAACGGGTTACGGGTC; AAATTAAGGGCAGGCCTTGGA). A standard curve was made using serial dilutions of control input DNA and regression analysis was used to calculate percent recovery (immunoprecipitated DNA/input DNA) × 100%.

#### Statistical analysis

Cohort sizes were determined by power analysis in R with the “pwr” package, using a Cohen’s d effect size calculated from previous experiments of cortisol measurements in adult zebrafish treated as larvae with 1 uM cortisol. This analysis indicated that a minimum n of 5 is needed to detect statistically significant (p < 0.05) differences between treatment and controls. Some experiments had multiple biological replicates, whereas others were done only once with technical replicates, as noted in the Figure legends. Tissue cortisol measurements in Fig. 1b were analyzed with three-way ANOVA to assess the interactions of tissue type, fed/fasted state, and larval cortisol treatment on adult tissue cortisol level. Post-hoc two-way ANOVA was then performed for each tissue type. Grand means in Fig. 2b were assessed with a two-tailed *t* test.

## Limitations

- The measurements shown in Fig. 1 were done only once.
- When interpreting the single time point measurements reported here it is important to consider that cortisol levels are dynamic, with circadian rhythms as well as ultradian fluctuations with an order of magnitude dynamic range.

## Supplementary information

**Additional file 1:** ANOVA of tissue cortisol levels shown in Fig. 1b.

**Additional file 2: Figure S1.** Top ten transcription factor binding motifs identified by HOMER motif enrichment analysis of sequences from the 251 peaks that scored ≥ 100.

**Additional file 3: Figure S2.** Survey of gene expression by 96-well qPCR array and relative expression levels of *klf9* and *fkbp5* in brain tissue dissected from different experimental cohorts. Each measurement was made from pooled brain RNA of 6 individuals of mixed sex (equal representation), except where indicated (panel **f**). The qPCR measurement shown in panel (**b**) is from the same RNA samples that were used to generate the data depicted in panel (**a**).

**Additional file 4: Methods.** ATAC-seq library construction protocol, sequencing and read processing.

## Data Availability

The ATAC-seq dataset generated during this study is available in the NCBI Gene Expression Omnibus (GEO) repository, under accession number GSE137987.

## Abbreviations

ELS: Early life stress
GC: Glucocorticoid
GR: Glucocorticoid receptor
ATAC-seq: Assay for transposase accessible chromatin–sequencing
qRT-PCR: Quantitative reverse transcription and polymerase chain reaction
ELISA: Enzyme linked immunosorbant assay
