# Supplementary material for: Chronic cortisol exposure in early development leads to neuroendocrine dysregulation in adulthood
Source: BMC Res Notes. 2020 Aug 3;13:366. doi: 10.1186/s13104-020-05208-w (PMC7398215; doi:10.1186/s13104-020-05208-w)
Supplement: Supplementary file 1 — Additional file 1: ANOVA of tissue cortisol levels shown in Fig. 1b. [file 13104_2020_5208_MOESM1_ESM.html]

3-Way & 2-Way ANOVA on Zebrafish Tissue Cortisol Data


# 3-Way & 2-Way ANOVA on Zebrafish Tissue Cortisol Data

#### Elli Hartig

#### 5/3/2020

# Three-Way Anova

To assess the effects of independent variables: tissue type, fed/fasted state, and larval cortisol treatment on dependent variable cortisol level:

```
#import cortisol ELISA data
zf_cortisol <- read.table("zebrafish_cortisol/fedstate_cortisol_data.txt", header = TRUE)

zf_cortisol_techreps <- read.table("zebrafish_cortisol/fedstate_cortisol_data_technicalreplicates.txt", header = TRUE)

#change larval_cort from numeric to factor

zf_cortisol$larval_cort <- factor(zf_cortisol$larval_cort,
                                  levels = c(0,1))

zf_cortisol_techreps$larval_cort <- factor(zf_cortisol$larval_cort,
                                  levels = c(0,1))
#Run 3-way Anova to assess variance of dependent variable (cort_level) across independent variables tissue, fed/fasted state, and larval cortisol exposure

res.aov3 <- aov(cort_level ~ tissue * fed_state + larval_cort, data = zf_cortisol)
summary(res.aov3)
```

```
##                  Df Sum Sq Mean Sq F value  Pr(>F)   
## tissue            5   3867   773.5   6.347 0.00523 **
## fed_state         1     38    37.7   0.310 0.58912   
## larval_cort       1    267   266.7   2.189 0.16709   
## tissue:fed_state  5   2205   440.9   3.618 0.03517 * 
## Residuals        11   1340   121.9                   
## ---
## Signif. codes:  0 '***' 0.001 '**' 0.01 '*' 0.05 '.' 0.1 ' ' 1
```

Cortisol level is significantly different across tissues with a p-value of 0.00523.

There is also a significant interaction affect of tissue/fed state, with a p-value of 0.03517.

However, there is not a significant, concerted effect of larval cortisol exposure across tissues and fed states.

### Checking ANOVA assumptions

Verify the distribution of residuals is normal:

```
res <- res.aov3$residuals
hist(res, main = "3-Way Anova Residuals")
```

# Tissue-specific Two-Way ANOVAs

Unfortunately, because there is only 1 biological replicate (pooled), I will have to use the technical replicates for the individual tissue ANOVA tests.

### Kidney

```
kidney_cortisol <- filter(zf_cortisol_techreps, tissue == "KIDNEY")

res.aov2.kidney <- aov(cort_level ~ larval_cort * fed_state, data = kidney_cortisol)
summary(res.aov2.kidney)
```

```
##                       Df Sum Sq Mean Sq F value Pr(>F)  
## larval_cort            1     43    43.2   0.087 0.7752  
## fed_state              1   2681  2681.4   5.416 0.0484 *
## larval_cort:fed_state  1    426   426.3   0.861 0.3806  
## Residuals              8   3961   495.1                 
## ---
## Signif. codes:  0 '***' 0.001 '**' 0.01 '*' 0.05 '.' 0.1 ' ' 1
```

### Blood

```
blood_cortisol <- filter(zf_cortisol_techreps, tissue == "BLOOD")

res.aov2.blood <- aov(cort_level ~ larval_cort * fed_state, data = blood_cortisol)
summary(res.aov2.blood)
```

```
##                       Df Sum Sq Mean Sq F value   Pr(>F)    
## larval_cort            1     21      21   0.488    0.505    
## fed_state              1   3987    3987  92.204 1.15e-05 ***
## larval_cort:fed_state  1     55      55   1.272    0.292    
## Residuals              8    346      43                     
## ---
## Signif. codes:  0 '***' 0.001 '**' 0.01 '*' 0.05 '.' 0.1 ' ' 1
```

### Brain

```
brain_cortisol <- filter(zf_cortisol_techreps, tissue == "BRAIN")

res.aov2.blood <- aov(cort_level ~ larval_cort * fed_state, data = blood_cortisol)
summary(res.aov2.blood)
```

```
##                       Df Sum Sq Mean Sq F value   Pr(>F)    
## larval_cort            1     21      21   0.488    0.505    
## fed_state              1   3987    3987  92.204 1.15e-05 ***
## larval_cort:fed_state  1     55      55   1.272    0.292    
## Residuals              8    346      43                     
## ---
## Signif. codes:  0 '***' 0.001 '**' 0.01 '*' 0.05 '.' 0.1 ' ' 1
```

### Gut

```
gut_cortisol <- filter(zf_cortisol_techreps, tissue == "GUT")

res.aov2.gut <- aov(cort_level ~ larval_cort * fed_state, data = gut_cortisol)
summary(res.aov2.gut)
```

```
##                       Df Sum Sq Mean Sq F value Pr(>F)
## larval_cort            1  0.914   0.914   0.397  0.546
## fed_state              1  6.062   6.062   2.628  0.144
## larval_cort:fed_state  1  0.150   0.150   0.065  0.805
## Residuals              8 18.451   2.306
```

### Skin

```
skin_cortisol <- filter(zf_cortisol_techreps, tissue == "SKIN")

res.aov2.skin <- aov(cort_level ~ larval_cort * fed_state, data = skin_cortisol)
summary(res.aov2.skin)
```

```
##                       Df Sum Sq Mean Sq F value Pr(>F)  
## larval_cort            1   5.44   5.436   0.924 0.3645  
## fed_state              1  26.30  26.303   4.473 0.0674 .
## larval_cort:fed_state  1   2.98   2.984   0.507 0.4965  
## Residuals              8  47.05   5.881                 
## ---
## Signif. codes:  0 '***' 0.001 '**' 0.01 '*' 0.05 '.' 0.1 ' ' 1
```

### Muscle

```
muscle_cortisol <- filter(zf_cortisol_techreps, tissue == "MUSCLE")

res.aov2.muscle <- aov(cort_level ~ larval_cort * fed_state, data = muscle_cortisol)
summary(res.aov2.muscle)
```

```
##                       Df Sum Sq Mean Sq F value  Pr(>F)    
## larval_cort            1  0.648   0.648   4.169  0.0755 .  
## fed_state              1  8.942   8.942  57.506 6.4e-05 ***
## larval_cort:fed_state  1  0.224   0.224   1.439  0.2647    
## Residuals              8  1.244   0.155                    
## ---
## Signif. codes:  0 '***' 0.001 '**' 0.01 '*' 0.05 '.' 0.1 ' ' 1
```

Kidney, blood, brain, and muscle are all significantly effected by fed state with p < 0.05, but there is no significant, concerted effect of larval cortisol across fed state.

Overall, this supports our observation that the long-term effects of early-life cortisol treatment differ by tissue and are dynamic based on fed/fasted state.
