# Supplementary material for: Chronic cortisol exposure in early development leads to neuroendocrine dysregulation in adulthood
Source: BMC Res Notes. 2020 Aug 3;13:366. doi: 10.1186/s13104-020-05208-w (PMC7398215; doi:10.1186/s13104-020-05208-w)
Supplement: Supplementary file 2 — Additional file 2: Figure S1. Top ten transcription factor binding motifs identified by HOMER motif enrichment analysis of sequences from the 251 peaks that scored ≥ 100. [file 13104_2020_5208_MOESM2_ESM.pdf]

Rank	Motif	Name	P-value	q-value (Benjamini)	# Target Sequences with Motif	% Target Sequences with Motif	# Background Sequences with Motif	% Background Sequences with Motif
1	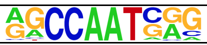	**NFY**(CCAAT)/Promoter/Homer	1e-83	0.0000	158	63.45	54161.2	11.54
2	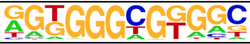	**KLF14**(Zf)/HEK293-KLF14.GFP-ChIP-Seq(GSE58341)/Homer	1e-23	0.0000	85	34.14	48970.8	10.44
3	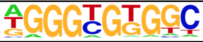	**KLF5**(Zf)/LoVo-KLF5-ChIP-Seq(GSE49402)/Homer	1e-20	0.0000	69	27.71	35600.4	7.59
4	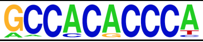	**Klf4**(Zf)/mES-Klf4-ChIP-Seq(GSE11431)/Homer	1e-15	0.0000	32	12.85	9880.1	2.11
5	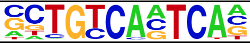	**Pknox1**(Homeobox)/ES-Prep1-ChIP-Seq(GSE63282)/Homer	1e-13	0.0000	35	14.06	13867.1	2.96
6	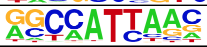	**Nanog**(Homeobox)/mES-Nanog-ChIP-Seq(GSE11724)/Homer	1e-12	0.0000	173	69.48	219576	46.8
7	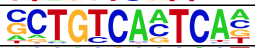	**Pbx3**(Homeobox)/GM12878-PBX3-ChIP-Seq(GSE32465)/Homer	1e-12	0.0000	34	13.65	14352.2	3.06
8	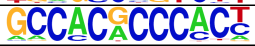	**Klf9**(Zf)/GBM-Klf9-ChIP-Seq(GSE62211)/Homer	1e-11	0.0000	29	11.65	10668.2	2.27
9	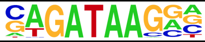	**Gata1**(Zf)/K562-GATA1-ChIP-Seq(GSE18829)/Homer	1e-9	0.0000	40	16.06	23981.9	5.11
10	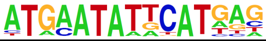	**OCT**:OCT(POU,Homeobox)/NPC-Brn1-ChIP-Seq(GSE35496)/Homer	1e-9	0.0000	10	4.02	966.8	0.21
