# Supplementary figures and images for: Chronic cortisol exposure in early development leads to neuroendocrine dysregulation in adulthood
Source: BMC Res Notes. 2020 Aug 3;13:366. doi: 10.1186/s13104-020-05208-w (PMC7398215; doi:10.1186/s13104-020-05208-w)

**A**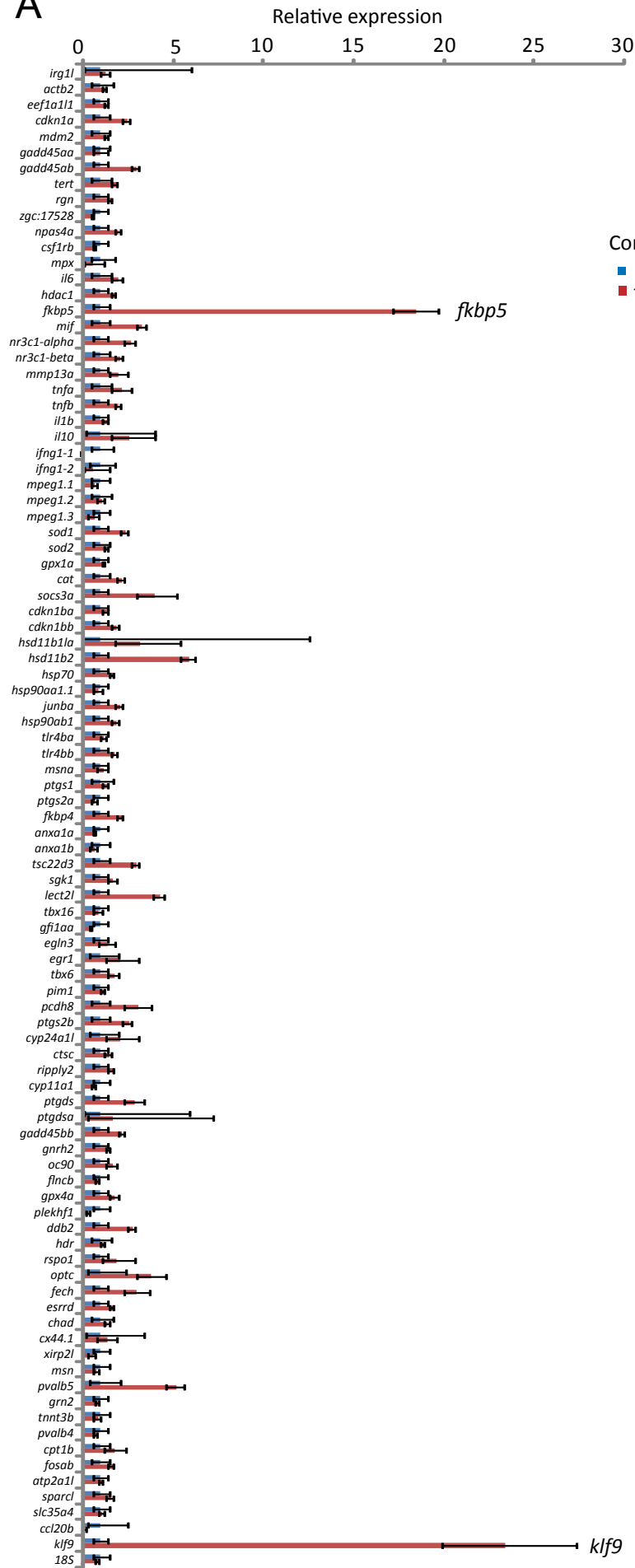**B**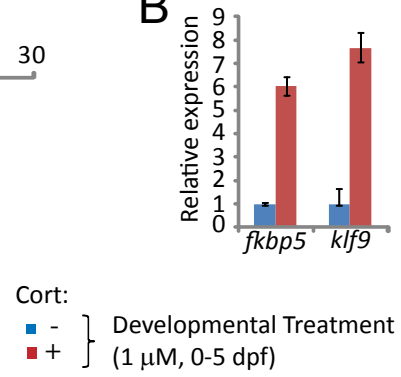**C**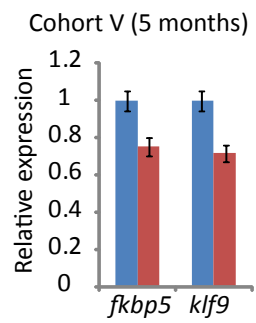**D**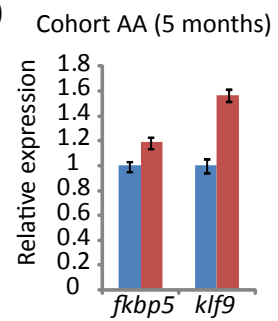**E**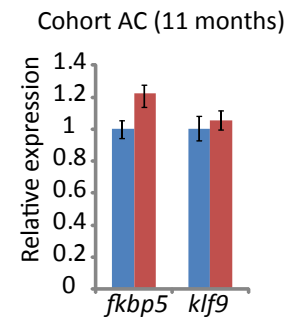**F**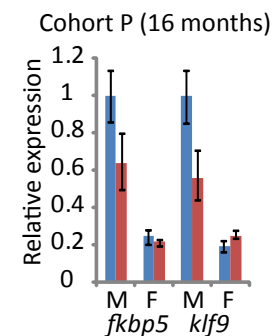

Supplement: Supplementary file 3 — Additional file 3: Figure S2. Survey of gene expression by 96-well qPCR array and relative expression levels of klf9 and fkbp5 in brain tissue dissected from different experimental cohorts. Each measurement was made from pooled brain RNA of 6 individuals of mixed sex (equal representation), except where indicated (panel f). The qPCR measurement shown in panel (b) is from the same RNA samples that were used to generate the data depicted in panel (a). [file 13104_2020_5208_MOESM3_ESM.pdf]
