# Supplementary material for: Chronic cortisol exposure in early development leads to neuroendocrine dysregulation in adulthood
Source: BMC Res Notes. 2020 Aug 3;13:366. doi: 10.1186/s13104-020-05208-w (PMC7398215; doi:10.1186/s13104-020-05208-w)
Supplement: Supplementary file 4 — Additional file 4: Methods. ATAC-seq library construction protocol, sequencing and read processing. [file 13104_2020_5208_MOESM4_ESM.docx]

**Chronic cortisol exposure in early development leads to neuroendocrine dysregulation in adulthood**

Ellen I. Hartig, Shusen Zhu, Benjamin L. King, and James A. Coffman

**Additional methods**

**ATAC-seq library construction**

ATAC-seq libraries were constructed using the Nextera DNA Library Prep kit (Illumina Cat #FC-121-1030), following the procedures provided with the kit with minor modifications:

**Materials**

Phosphate Buffered Saline (PBS)

Molecular biology grade IGEPAL CA-630

Lysis buffer (10 mM Tris-HCl, pH 7.4, 10 mM NaCl, 3 mM MgCl2, 0.1% IGEPAL CA-630)

Nextera DNA Library Prep kit (Illumina Cat #FC-121-1030, 24 samples)

NPM (Nextera PCR Master Mix)

PPC (PCR Primer Cocktail)

RSB (Resuspension Buffer)

TD (Tagment DNA Buffer, or 2x reaction buffer)

TDE1 (Tagment DNA Enzyme, or Nextera Tn5 Transposase)

Qiagen MinElute PCR Purification Kit

Index 1 (i7) Adapters

Index 2 (i5) Adapters

20 x Evagreen

0.2-ml PCR tubes

PCR Thermal cycler

***Note:*** *When ordering primers for use in Illumina libraries, make certain to include the modifications (e.g. 5’-phosphorylation and phosphorothioate bonds on the 3' terminal nucleotide) and ensure the oligos are PAGE purified. Even small amounts of n-1 primers will lead to messy out-of-phase sequencing and cause clusters to fail filtering.*

**Cell Preparation**

1. Dilute blood cells (3x10^6^ cells/µl for zebrafish) with PBS and take 200,000 cells.

Cells should be intact and in a homogenous single cell suspension.

2. Spin down 200,000 cells at 500 ×g for 5 min, 4°C.

The number of cells at this step is crucial as the transposase to cell ratio sets the distribution of DNA fragments generated.

3. Gently pipette to resuspend the cell pellet in 50 μl of cold lysis buffer. Spin down immediately at 500 ×g for 10 min, 4°C.

This step provides lysis of cells with non-ionic detergent and generates of a crude nuclei preparation.

4. Discard the supernatant, and immediately continue to transposition reaction.

**Transposition Reaction and Purification**

1. Keep the cell pellet on ice.

Thaw TD and TDE1 on ice. Gently invert the thawed tubes 3–5 times, and then centrifuge briefly.

2. To make the transposition reaction mix, combine the following:

25 μl TD (2x reaction buffer)

5μl TDE1 (Nextera Tn5 Transposase)

20μl Nuclease Free H2O

3. Resuspend nuclei in the transposition reaction mix.

4. Incubate the transposition reaction at 55°C for 6 min.

Mix gently to increase fragment yield.

5. Immediately following transposition, purify using a Qiagen MinElute PCR Purification Kit.

6. Elute transposed DNA in 10 μl Elution Buffer (10 mM Tris buffer, pH 8).

7. Purified DNA can be stored at −20°C.

This is a convenient stopping point. Note that the DNA fragments are not PCR amplifiable if melted at this point.

**PCR Amplification**

1. Thaw Index adapters (i7 and i5), NPM and PPC on ice for ~20 minutes. Gently invert the thawed tubes 3–5 times, and then centrifuge briefly.

To amplify transposed DNA fragments, combine the following in a 0.2 ml PCR tube:

10 μl Transposed DNA

10 μl Nuclease Free H2O

5μl Index 1 (i7) adapter

5μl Index 2 (i5) adapter

5 μl PPC

15 μlNPM

2. Thermal cycle as follows:

1 cycle of 72°C for 5 min, 98°C for 30 sec

5 cycles of 98°C for 10 sec, 63°C for 30 sec, 72°C for 3 min

Hold at 10°C.

This first 5-minute extension at 72°C is critical to allow extension of both ends of the primer after transposition, thereby generating amplifiable fragments. This short pre-amplification step ensures that downstream quantitative PCR (qPCR) quantification will not change the complexity of the original library.

3. To reduce GC and size bias in PCR, the appropriate number of PCR cycles is determined using qPCR allowing us to stop amplification prior to saturation. To run a qPCR side reaction, combine the following in qPCR compatible consumables:

5 μl of previously PCR amplified DNA

4.75μl Nuclease Free H2O

0.5 μl Index 1 (i7) adapter

0.5 μl Index 2 (i5) adapter

0.5 μl PPC

0.75 μl 20x Evagreen

3 μl NPM

4. Using a qPCR instrument, cycle as follows:

1 cycle of 98°C for 30 sec

20 cycles of 98°C for 10 sec, 63°C for 30 sec, 72°C for 3 min

5. To calculate the additional number of cycles needed, plot linear Rn versus cycle and determine the cycle number that corresponds to 1/4 ~ 1/3 of maximum fluorescent intensity.

The purpose of this qPCR step is to generate libraries that are minimally PCR amplified. Most PCR bias comes from later PCR cycles that occur during limited reagent concentrations. This determination of the optimal number of cycles to amplify the library reduces artifacts associated with saturation PCR of complex libraries.

6. Run the remaining 45 μl PCR reaction to the cycle number determined by qPCR. Cycle as follows:

1 cycle of 98°C for 30 sec

N cycles of 98°C for 10 sec, 63°C for 30 sec, 72°C for 3 min

Cycle for an additional N cycles, where N is determined using qPCR.

**Optional library quality control using gel electrophoresis**

For assessing quality, amplified libraries can be visualized using gel electrophoresis prior to PCR purification. The low concentration of the amplified materials requires a 5% TBE polyacrylamide gel optimized for sensitivity. We load 50 ng of 100-bp DNA ladder from NEB and 5 μl of the PCR products on the gel. Run at ~100 mV for 3 hours. Then stain the gel with 1xSYBR safe in 0.6xTBE buffer for 30 min.

Typical libraries show a broad size distribution from ~250 bp to 1000 bp with average library size of 500 bp. Fragment sizes as small as 250 bp to as large as 1000 ~ 1500 bp can be sequenced.

7. Purify amplified library using Qiagen MinElute PCR Purification Kit. Elute the purified library in 20 μl Elution Buffer (10 mM Tris Buffer, pH 8). Be sure to dry the column before adding elution buffer.

The concentration of DNA eluted from the column ought to be approximately 30 nM, however 5-fold variation is possible and not detrimental.

**Sequencing and processing of ATAC-seq sequence reads**

Libraries were sequenced at the Jackson Laboratory NextGen Sequencing Facility (Bar Harbor Maine) using an Illumina NextSeq 500 (Illumina, San Diego, CA).

Following initial diagnostic sequence analyses using FastQC (v0.11.8; <https://www.bioinformatics.babraham.ac.uk/projects/fastqc/)>, reads were trimmed using Trimmomatic (v0.32) [1] with parameters ‘ILLUMINACLIP:TruSeq3-PE.fa:2:30:10 LEADING:3 TRAILING:3 SLIDINGWINDOW:4:15 MINLEN:36’. The remaining paired reads were truncated to 30bp reads using fastx_trimmer from the FASTX Toolkit (v0.0.13; http://hannonlab.cshl.edu/fastx_toolkit/) with parameters ‘-Q33 -l 30’. Truncated reads were then mapped to the zebrafish GRCz10 genome assembly (RefSeq assembly accession: GCF_000002035.5) using bowtie (v1.0.0) [2] with parameters ‘--chunkmds 520 -y -v 2 --best --strata -m 3 -k 1 -S -p 8’. Read alignments to the mitochondrial genome were filtered out from the SAM files and the resulting SAM files were converted to BAM files using samtools (v0.1.18) [3]. Nuclease accessible peaks were called using the callpeak function in MACS2 (v2.1.1.20160309) [4] with parameters ‘-f BAM -g 146444456 -nomodel -nolabbda -keep-dup all -call-summits -q 0.001 –B’ and Ensembl (v85) [5] annotation of GRCz10. Differential binding events were called using the bdgdiff function in MACS2 with parameters ‘ -C’ [6]. Motifs within peaks were predicted and annotated using HOMER (v4.8; <http://homer.ucsd.edu/home>) [7].

**References**

1. Bolger AM, Lohse M, Usadel B: **Trimmomatic: a flexible trimmer for Illumina sequence data**. *Bioinformatics* 2014, **30**(15):2114-2120.

2. Langmead B, Trapnell C, Pop M, Salzberg SL: **Ultrafast and memory-efficient alignment of short DNA sequences to the human genome**. *Genome Biol* 2009, **10**(3):R25.

3. Li H, Handsaker B, Wysoker A, Fennell T, Ruan J, Homer N, Marth G, Abecasis G, Durbin R: **The Sequence Alignment/Map format and SAMtools**. *Bioinformatics* 2009, **25**(16):2078-2079.

4. Zhang Y, Liu T, Meyer CA, Eeckhoute J, Johnson DS, Bernstein BE, Nusbaum C, Myers RM, Brown M, Li W *et al*: **Model-based analysis of ChIP-Seq (MACS)**. *Genome Biol* 2008, **9**(9):R137.

5. Yates A, Akanni W, Amode MR, Barrell D, Billis K, Carvalho-Silva D, Cummins C, Clapham P, Fitzgerald S, Gil L *et al*: **Ensembl 2016**. *Nucleic Acids Res* 2016, **44**(D1):D710-716.

6. Abbott BD, Birnbaum LS, Perdew GH: **Developmental expression of two members of a new class of transcription factors: I. Expression of aryl hydrocarbon receptor in the C57BL/6N mouse embryo**. *Dev Dyn* 1995, **204**(2):133-143.

7. Heinz S, Benner C, Spann N, Bertolino E, Lin YC, Laslo P, Cheng JX, Murre C, Singh H, Glass CK: **Simple combinations of lineage-determining transcription factors prime cis-regulatory elements required for macrophage and B cell identities**. *Mol Cell* 2010, **38**(4):576-589.
